# A neutralizing bispecific single-chain antibody against SARS-CoV-2 Omicron variant produced based on CR3022

**DOI:** 10.3389/fcimb.2023.1155293

**Published:** 2023-05-03

**Authors:** Kaikai Yu, Bin Liu, Haotian Yu, Chengbiao Sun, Xuefeng Wang, Guorui Li, Mingxin Dong, Yan Wang, Jianxu Zhang, Na Xu, Wensen Liu

**Affiliations:** ^1^ Changchun Veterinary Research Institute, Chinese Academy of Agricultural Science, Changchun, Jilin, China; ^2^ Academic Affairs Office, Jilin Medical University, Jilin, Jilin, China; ^3^ State Key Laboratory of Respiratory Disease, National Clinical Research Center for Respiratory Disease, Guangzhou Institute of Respiratory Health, The First Affiliated Hospital of Guangzhou Medical University, Guangzhou, Guangdong, China; ^4^ College of Life Sciences and Food Engineering, Inner Mongolia Minzu University, Tongliao, China

**Keywords:** SARS-CoV-2, COVID-19, single-chain variable fragment, bispecific antibody, Omicron variant

## Abstract

**Introduction:**

The constantly mutating SARS-CoV-2 has been infected an increasing number of people, hence the safe and efficacious treatment are urgently needed to combat the COVID-19 pandemic. Currently, neutralizing antibodies (Nabs), targeting the receptor-binding domain (RBD) of the SARS-CoV-2 spike protein are potentially effective therapeutics against COVID-19. As a new form of antibody, bispecific single chain antibodies (BscAbs) can be easily expressed in *E. coli* and exhibits broad-spectrum antiviral activity.

**Methods:**

In this study, we constructed two BscAbs 16-29, 16-3022 and three single chain variable fragments (scFv) S1-16, S2-29 and S3022 as a comparison to explore their antiviral activity against SARS-CoV-2. The affinity of the five antibodies was characterized by ELISA and SPR and the neutralizing activity of them was analyzed using pseudovirus or authentic virus neutralization assay. Bioinformatics and competitive ELISA methods were used to identify different epitopes on RBD.

**Results:**

Our results revealed the potent neutralizing activity of two BscAbs 16-29 and 16-3022 against SARS-CoV-2 original strain and Omicron variant infection. In addition, we also found that SARS-CoV RBD-targeted scFv S3022 could play a synergistic role with other SARS-CoV-2 RBD-targeted antibodies to enhance neutralizing activity in the form of a BscAb or in cocktail therapies.

**Discussion:**

This innovative approach offers a promising avenue for the development of subsequent antibody therapies against SARSCoV-2. Combining the advantages of cocktails and single-molecule strategies, BscAb therapy has the potential to be developed as an effective immunotherapeutic for clinical use to mitigate the ongoing pandemic.

## 1 Introduction

Coronavirus Disease 2019 (COVID-19), the first coronavirus pandemic in human history as declared by the World Health Organization (Bedford et al., 2020; Harcourt et al., 2020), has posed a serious threat to global public health, travel, and economy. The efficacy of existing COVID-19 vaccines (Koirala et al., 2020) and therapeutic antibodies approved worldwide were threatened due to the emergence of SARS-CoV-2 variants such as Delta variant and Omicron variant (Cherian et al., 2021; Lopez Bernal et al., 2021; Planas et al., 2021; Ren et al., 2022). Therefore, there is an urgent need for cost-effective and efficacious antibody-based therapeutics that potently neutralize variants and mitigate the COVID-19 pandemic.

SARS-CoV-2 and SARS-CoV (Genbank ID: AAS00002.1) are highly homologous and share about 86.15% identity with each other, both of which are enveloped, single-stranded, positive-sense RNA viruses with a genome of approximately 30,000 nucleotides in length (Zhou et al., 2020). The virus-encoded spike (S) protein of SARS-CoV-2, which belongs to the type I membrane fusion protein, consists of two subunits S1 and S2. The receptor binding domain (RBD) on S1 subunit can form homotrimers in different conformation and invade susceptible cells by binding to angiotensin converting enzyme 2 (ACE2) (Zhou et al., 2020). Recently, NAbs targeting the RBD of the SARS-CoV-2 spike protein are among the most promising approaches against COVID-19 (Altuntas et al., 2021; Rejeki et al., 2021) and have improved efficacy over convalescent plasma treatment (Klasse and Moore, 2020; Saif et al., 2020; Shen et al., 2020) which may cause antibody-dependent enhancement (ADE) (Chari, 2020). Bispecific single chain antibody (BscAb), the simplest structural form of bispecific antibody (BsAb) (Yang et al., 2017), are composed of two scFvs targeting different epitopes. The advantages of short development time, low production cost and high production efficiency make BscAbs a powerful means to defeat infectious disease pandemics (Tiller and Tessier, 2015). Combining the advantages of cocktails with single-molecule strategies, BscAbs have been widely used in cancer immunotherapy and antiviral therapy (Zhou et al., 2017; Wang et al., 2019).

Given those advantages, BscAbs have been identified as a promising alternative therapy against COVID-19. In this study, we developed and identified several high-affinity SARS-CoV-2 RBD targeted antibodies with desired neutralizing activities, including two BscAbs (16-29 and 16-3022) and three single-chain variable fragments (scFvs) (S1-16, S2-29, and S3022). The BscAbs and scFvs were constructed based on Nabs CoVA1-16, CoVA2-29, and CR3022, which were derived from donors who recovered from COVID-19 or SARS, respectively. (Brouwer et al., 2020; Yuan et al., 2020). S3022 is derived from Nab CR3022, which was originally isolated from a convalescent SARS patient (Marissen et al., 2006) and can target both the RBD of SARS-CoV-2 and the RBD of SARS-CoV as reported (Yuan et al., 2020). However, unlike most known SARS-CoV RBD-targeting antibodies (Berry et al., 2004; Sui et al., 2004; Marissen et al., 2006), the neutralization mechanism of CR3022 for SARS-CoV does not depend on direct blocking of receptor binding, which is consistent with the structural alignment that CR3022 does not compete with ACE2 for binding to the SARS-CoV-2 RBD (Lan et al., 2020). It has been shown that CR3022 can synergize with other RBD-targeting antibodies to neutralize SARS-CoV. However, whether CR3022 can synergize with other SARS-CoV-2 RBD-targeting antibodies remains to be determined. Therefore, we constructed BscAb 16-3022 to further explore the synergistic effect between S3022 and RBD-targeted scFv S1-16.

Our results demonstrated the effectiveness of the BscAbs in neutralizing SARS-CoV-2 and the Omicron variant. In addition, bioinformatics analysis revealed how the BscAbs target non-overlapping RBD epitopes with two independent Antigen binding sites, which further illustrated the potential of S3022 as a neutralizing activity enhancer working with other Nabs against SARS-CoV-2. BscAb therapy is likely to be developed as an effective bispecific immunotherapeutic for clinical application to mitigate the ongoing pandemic.

## 2 Materials and methods

### 2.1 Ethics

Antiviral study of BscAb or antibody cocktail against SARS-CoV-2 (BJ01) was performed at the Biosafety Level-3 Facilities at Key laboratory of Jilin Province for Zoonosis Prevention and Control under guidelines and protocols that were in line with institutional biosafety requirements. Antiviral study against SARS-CoV-2 Omicron variant was performed at the State Key Laboratory of Respiratory Disease in National Clinical Research Center for Respiratory Disease under guidelines and protocols that were in line with institutional biosafety requirements.

### 2.2 Gene construction

Five constructs, anti-SARS-CoV-2 scFv S1-16, S2-29 and S3022, BscAb 16-29 and 16-3022 were generated. To generate scFv S1-16, S2-29 and S3022, the variable region heavy and variable region light chain of neutralizing antibody CoVA1-16 Fab (Genebank: MT599919.1, MT599835.1), CoVA2-29 Fab (Genebank: MT599875.1, MT599959.1) and CR3022 Fab (PDB ID: 7A5R) were linked by a 15 amino acids (G4S)_3_ linker. BscAb 16-29 and 16-3022 were generated by linking scFv S1-16 and S2-29 or S3022 with another (G4S)_3_ linker. A prokaryotic expression vector pET-30a carried T7 strong promoter was used in this study, which exhibited high protein expression ability. Coding fragments were synthesized by Comate Bioscience Co., Ltd., and inserted between Nde1 and Xho1 multiple cloning site in the pET-30a vector with 6His-tag on C-terminal. The vector sequence and plasmid profile are shown in **Supplementary Figure 1**.

### 2.3 Protein expression and purification

All five constructs were expressed as inclusion body in E. coli and purified on Ni-NTA column. After purification, protein renaturation was performed to gain active antibodies. Briefly, the five expression vectors were transformed into *E.coli* strain BL21 (DE3) competent cells (TransGen Biotech, Beijing, China). A single round colony was inoculated into 20 mL of LB medium containing 100 µg/mL kanamycin and incubated at 37°C 180 rpm for 5 h. The incubated culture was transferred to 2 L of LB medium with 100 µg/mL kanamycin for large scale culture for 4–6 h at 37°C. Then, 0.5 mM isopropyl-β-d-thiogalactoside (IPTG) was applied to induce protein overexpression when the OD value was above 0.6, and the cells were grown overnight at 37°C before harvesting. Then, the culture was centrifuged at 8000 rpm for 30 min at 4°C. Precipitation was collected and resuspend with PBS, then the resuspending was homogenized using a high pressure homogenizer. The precipitation after centrifugation was washed twice with inclusion body purgation buffer and once with PBS. Inclusion bodies were dissolved with 8 M urea after purification by Ni Sepharose 6FF (GE Healthcare, Little Chalfont, Buckinghamshire, UK). After denaturing with urea and gradient dialysis, refolded scFvs and BscAbs were obtained in solution with 50 mM disodium hydrogen phosphate (pH=8.0). The proteins were resolved with SDS-PAGE and stained using a solution of Coomassie blue R-250. The purified protein concentration was measured using the Micro BCA Protein Assay Kit (Beyotime, Shanghai, China).

### 2.4 ELISA analysis

Indirect ELISA was carried out to measure the affinities between antibodies and SARS-CoV-2 RBD. Mouse Fc-fused SARS-CoV-2 RBD (Sino Biological, Beijing, China) or PBS (false positive control) was coated onto a 96-well ELISA plate at a concentration of 0.5 μg/mL in coating buffer (20 mM PBS, pH=7.4) overnight at 4°C and was blocked with a blocking buffer (3% PBS-BSA) at room temperature for 2 h. After three times of washing with PBST (PBS, 0.05% Tween 20), antibodies were serially diluted in the PBS, starting from 12 µM to 0.023 µM, and 100 µL of each concentration was addition to the RBD-coated plates as primary antibody for 2 h. Substitution of PBS (pH=7.2) for the primary antibody served as the negative control. HRP-conjugated rabbit anti-6×His secondary antibodies (Thermo Fisher Scientific, Waltham, MA, USA) were diluted 1:10000 and incubated with each well for 1 h at 37°C. After five times of washing with PBST, the samples were further incubated with TMB substrate solution 100 μL per well for 10 min to develop the signals. After the addition of stop solution (2 M H_2_SO_4_) 100 μL per well for the termination of the reaction, the absorbance was measured at 450 nm using a microplate reader. For competitive ELISA, S3022 was coated onto a 96-well ELISA plate at a concentration of 10 μg/mL, the mixture of scFv at 10 μg/mL and Fc-fused SARS-CoV-2 RBD (Sino Biological, Beijing, China) at 100 ng/mL were used as primary antibody, and HRP-conjugated goat-anti-mouse IgG (Thermo Fisher Scientific, Waltham, MA, USA) was used as secondary antibody. The remaining steps are the same as above.

### 2.5 Western blot analysis

To identify the binding of the antibodies and SARS-CoV-2 RBD, purified antibodies were resolved using SDS-PAGE, and then transferred to PVDF membranes (GE Healthcare, Little Chalfont, Buckinghamshire, UK). After that, the membranes were blocked with 3% PBS-BSA for 1 h at room temperature and incubated with 0.5 μg/mL mouse Fc-fused SARS-CoV-2 RBD (Sino Biological, Beijing, China) served as primary antibody at 4°C overnight. After three times of washing with PBST, membranes were incubated with 1:40000 dilution HRP-conjugated goat-anti-rabbit IgG (Thermo Fisher Scientific, Waltham, MA, USA) secondary antibody for 1h. The protein bands were visualized using an enhanced chemiluminescence detection system (Bio-Rad, Hercules, CA, USA).

### 2.6 SPR

The SPR was performed at room temperature using a BiaCore T200 with CM5 sensor chips (GE Healthcare).The 1:1 mixture of 0.1 M NHS (N-hydroxysuccinimide) and 0.1 M EDC (3-(N, N-dimethylamino) propyl-N-ethylcarbodiimide) were added on the surfaces of the sample and reference flow cells for activation at a flow rate of 10 μL/min. The reference flow cells were left blank. Ethanolamine was used to block all the surfaces (1 M, pH=8.0). HBS-EP (0.01 M HEPES, 150 mM NaCl, 3 mM EDTA, 0.05% surfactant TWEEN 20, pH 7.4) was used as running buffer. For binding affinity assays, the mouse Fc-fused SARS-CoV-2 RBD was diluted in 10 mM sodium acetate buffer (pH=5.5) and then immobilized on the CM5 sensor chip at about 300 response units. Antibodies S1-16, S2-29, S3022, 16-29, and 16-3022 at gradient concentrations (0, 7.8125 nM, 15.625 nM, 31.25 nM, 62.5 nM, 125 nM, 250 nM) were flowed over the chip surface. The sensor surface was regenerated with 10 mM glycine-HCl (pH=2.5) after each cycle. A Biacore T200 Evaluation software was used to analyze the date and fitted to the 1:1 interaction model.

### 2.7 Cell viability assay

Vero E6 cells were seeded (5×10^4^cells per well) in 96-well plates (Costar, Cambridge, MA, USA) and incubated for 24 h at 37°C in a 5% CO_2_ atmosphere. Then, antibodies were applied at various concentrations ranging from 1µM to 10 µM and the cells were incubated for 48 h at 37°C. 20 µL MTT solution (Intron, Seongnam, Korea) was added to each well, and the cells were incubated at 37°C for 4 h. After removing the supernatant, DMSO (150 µL) was added and cellular viability was measured at 492 nm using a microplate reader.

### 2.8 Pseudotyped virus neutralization assay

The pseudotyped virus neutralization activity of the antibodies was measured as previously reported (Wang et al., 2019; De Gasparo et al., 2021). In brief, the SARS-CoV-2 pseudovirus was prepared by co-transfecting HEK293T cells with plasmids encoding an env-defective, luciferase-expressing HIV-1 genome (pNL4-3.luc.RE) and the pCAGGS-SARS-CoV-2-S expression plasmid using Lipofectamine 2000 (Invitrogen, Carlsbad, CA, USA). The supernatant containing pseudovirus was harvested 48 h following transfection and frozen at -80°C for long-term storage after clarifying by centrifugation and filtering through a 0.45 μm sterilized membrane. Pseudovirus neutralizing assay was carried out as follows. Briefly, pseudotyped virus containing supernatants were incubated with threefold serially diluted antibodies at 37°C for 1h, before incubation with pre-plated target cells in 96-well plates at the density of 10^4^ cells/well. Vero cells without pseudovirus and media without antibody were used as positive and negative controls, respectively. Cells were lysed using cell lysis buffer (Promega, Madison, WI, USA) at 48 h and the lysates were transferred into 96-well luminometer plates after the cells re-feded with fresh medium at 4 h. Luciferase substrate (Promega, Madison, WI, USA) was added to the plates, then the relative luciferase activity was determined representing the inhibition degree of pseudotyped. Statistical analysis was performed using GraphPad Prism 8 to calculate IC_90_.

### 2.9 RNA isolation and reverse transcriptase-quantitative PCR analysis

Assays for determining the 50% tissue culture infectious dose (TCID_50_) with authentic SARS-CoV-2 (BJ01, accession number: MT291831) were performed using Vero cells. Briefly, Vero cells were seeded in a 24-well plate 24 h before infection at a density of 8×10^4^ cells/well. Cells were fixed and stained with crystal violet after 72 h of incubation with 10-fold serially diluted viruses and the media removement. The TCID_50_/ml titer was then determined. The measurement of the viral genome copy number was performed as previously reported (De Gasparo et al., 2021). In brief, Vero cells were grown in 96-well plates 1 day prior to infection. Indicated concentrations of antibodies or mock-treated with Dulbecco’s Modified Eagle Medium (Corning Incorporated, NY, NY, USA) supplemented with 10% FBS and penicillin-streptomycin were mixed with SARS-CoV-2 at 100 TCID_50_ (Yang et al., 2022) per well and incubated on Vero cells at 37°C for 1 h under 5% CO_2_. After washing the cells with fresh medium, the mixture was incubated for 24 h at 37°C. The culture supernatants were collected for RT-qPCR. Viral RNA in the supernatant was extracted using a Magnetic Viral DNA/RNA Kit (Tiangen, Beijing, China) following the manufacturer’s instructions. RNA genome copy numbers were identified with a 2019-nCoV Nucleic Acid Detection Kit using an RT-PCR fluorescence probe (Puruikang, Wuxi, China). RT-qPCR analysis for SARS-CoV-2 Omicron variant (BA.5, isolated from the First Affiliated Hospital of Guangzhou Medical University) was performed using the methods described above. The relative mRNA expression was calculated using the 2^−△△Ct^ method with GAPDH as an internal reference gene.

### 2.10 Computerized simulation to predict the RBD residues bound by S1-16, S2-29, and S3022

The crystal structure of SARS-CoV-2 RBD in the PDB protein database (ID: 6M0J) and the crystal structure of CoVA1-16 Fab (ID: 7JMX) and CR3022 Fab (ID: 7A5R) were used as templates to optimize and construct the three-dimensional structure of SARS-CoV-2 RBD and scFvs. The homologous modeling of the antibodies and SARS-CoV-2 RBD was performed on Swiss-Model online platform at https://swissmodel.expasy.org/. The modeled 3D protein structures were subsequently analyzed using Discovery Studio 4.5 (Acclery, Inc.). After the ZDOCK program, the optimized structure based on the CHARMM energy minimization method took the top 30 constellations with the highest ZDOCK scores and the top one was chosen. The molecular interactions were calculated using the ZDOCK algorithm after the visualization of the 3D protein structures in Discovery Studio Client. The molecular docking results were analyzed, and some key amino acid residues for antibody interaction with SARS-CoV-2 RBD were selected and displayed.

### 2.11 Statistical analysis

Statistical analysis of data were performed using GraphPad Prism 8. Values are expressed in graph bars as mean ± SD of at least three independent experiments and a p-value < 0.05 was considered statistically significant. Significance was indicated as follows: *p* ≤ 0.05 (*); *p* ≤ 0.01 (**); *p* ≤ 0.001 (***); *p* ≤ 0.0001 (****). The data comparison between the two groups was analyzed by the T test, while the comparison among multiple groups was surveyed with Ordinary one-way ANOVA.

## 3 Results

### 3.1 Expression and purification of anti-SARS-CoV-2 scFvs and BscAbs

**Figure 1:**
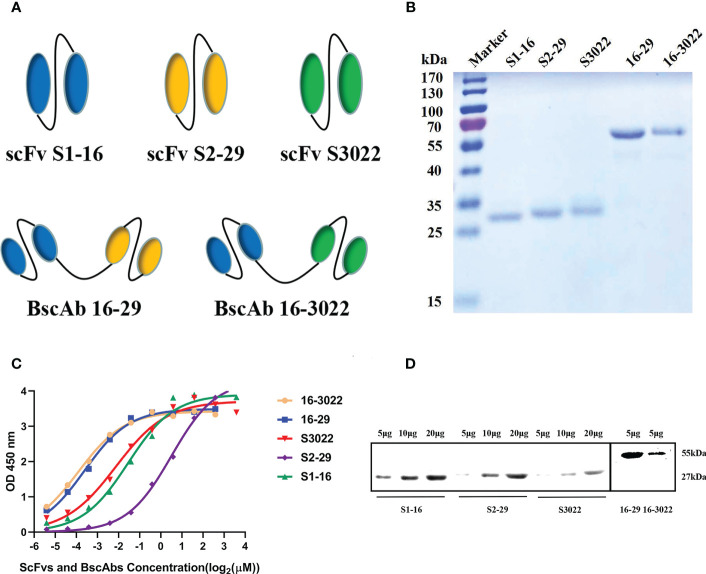
Construction and characterization of anti-SARS-CoV-2 scFvs and BscAbs. **(A)** Schematic structure of scFv S1-16, S2-29, S3022 and BscAb 16-29 and 16-3022; **(B)** The purified recombinant proteins of scFvs and BscAbs were separated by SDS-PAGE and stained with Coomassie Blue; **(C)** ScFvs and BscAbs binding to SARS-CoV-2 RBD measured by ELISA. Two-fold serial diluted scFvs from 12 μM or two-fold serial diluted BscAbs from 6 μM showed an dose-dependent binding to SARS-CoV-2 RBD; **(D)** Western blot analyses of scFvs and BscAbs incubated with mouse Fc-fused SARS-CoV-2 RBD. Each concentration in the left image above represents the mean value of three separate experiments.

Five expression vectors were constructed and the sequences of BscAbs were composed of two different scFv sequences linked by a (G_4_S)_3_ linker. The linker between two scFvs can result in the formation of BscAbs with two VHs and VLs (**Figure 1A**). All five proteins were expressed in *E. coli* cells in a form of inclusion body and purified using a Ni-NTA column. The refolded proteins were analyzed on a 12.5% SDS-PAGE gel (**Figure 1B**). The scFvs obtained in this study have an average molecular weight of approximately 27 kDa and a length of about 250 amino acids, while the BscAbs have a molecular weight of about 55 kDa (**Figure 1B**).

### 13.2 The identification of scFvs and BscAbs binding to SARS-CoV-2 RBD

To investigate the interaction between antibodies and SARS-CoV-2 RBD, enzyme linked immunosorbent assay (ELISA) and Western Blot assay were performed. For the ELISA assay, double serially diluted antibodies at the concentration ranging from 12 μM to 23 nM were used to detect solid-coated RBD, and the results showed a dose-dependent binding between antibodies and RBD (**Figure 1C**). BscAbs exhibited superior antigen-binding properties than scFvs (**Figure 1C**) because of the increased number of variable regions. Consistent with ELISA results, Western blot assay showed that the 5μg scFvs or BscAbs could specifically bound to RBD (**Figure 1D**).

### 3.3 Affinity measurement of the antibodies

**Figure 2:**
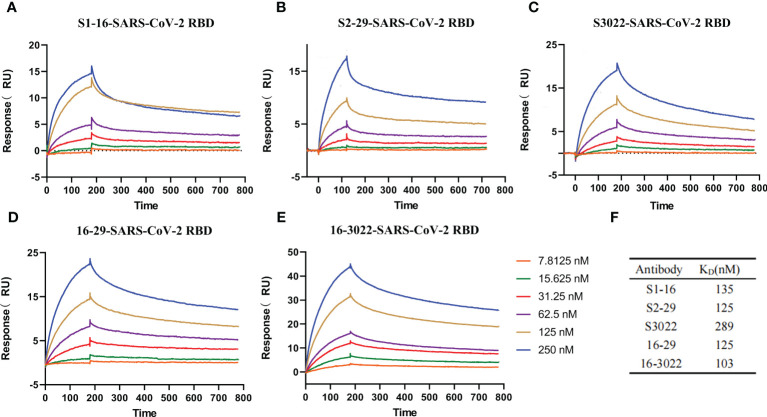
Affinity measurement of the antibodies. **(A–E)** Five antibodies binding to SARS-CoV-2 RBD measured by SPR. Two-fold serial dilutions antibodies from 250 nM to 7.8125 nM injected onto the captured RBD protein. Kinetic data from one representative experiment were fit to a 1:1 binding model. The profiles are shown for S1-16 **(A)**, S2-29 **(B)**, S3022 **(C)**, 16-29 **(D)**, and 16-3022 **(E)**. **(F)** Summary of SPR affinity measurements. The equilibrium dissociation constant (KD) are presented. Each concentration in the image above represents the mean value of three separate experiments.

Surface plasmon resonance (SPR) technology was utilized to characterize the interactions between SARS-CoV-2 RBD and antibodies. To determine the affinity constants of SARS-CoV-2 RBD binding to purified scFvs and BscAbs, SPR was carried out as a golden standard. Various concentrations of purified scFvs or BscAbs were prepared and injected to pass over the surface after the SARS-CoV-2 RBD immobilizing on the surface of Biacore Chip CM5. SPR results revealed that the equilibrium dissociation constant (KD) for the SARS-CoV-2 RBD against scFvs S1-16, S2-29 and S3022 was 135 nM, 125 nM and 289 nM, respectively (**Figures 2A–C**, **F**). And the KD for BscAbs 16-29 and 16-3022 was 125 nM and 103 nM (**Figures 2D–F**). Consistent with the indirect ELISA results, BscAbs exhibited better affinity activity than scFvs. Overall, the scFvs and BscAbs identified in this study revealed a potential binding performance in a SARS-CoV-2 specific manner.

### 3.4 Neutralizing of SARS-CoV-2 pseudovirus by BscAbs or a cocktail of scFvs

**Figure 3:**
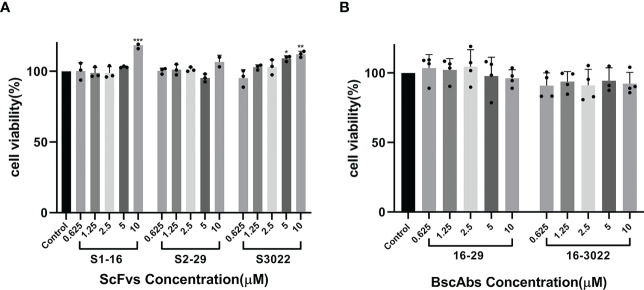
Cytotoxicity testing of scFvs and BscAbs in Vero E6 cells by MTT assay. **(A)** The effect of two-fold serial diluted scFvs from 10 μM on Vero E6 cells growth were evaluated; **(B)** The same dilution of BscAbs on Vero E6 cells growth was evaluated. Each concentration in the image above represents the mean value of three separate experiments. p ≤ 0.05 (*); p ≤ 0.01 (**); p ≤ 0.001 (***); p ≤ 0.0001(****).

The cell viability after scFvs or BscAbs treatment was determined by MTT assay in Vero E6 cells. Both scFvs and BscAbs did not show cytotoxicity in Vero E6 cells at concentrations ranging from 1 µM to 10 µM (**Figures 3A, B**).

**Figure 4:**
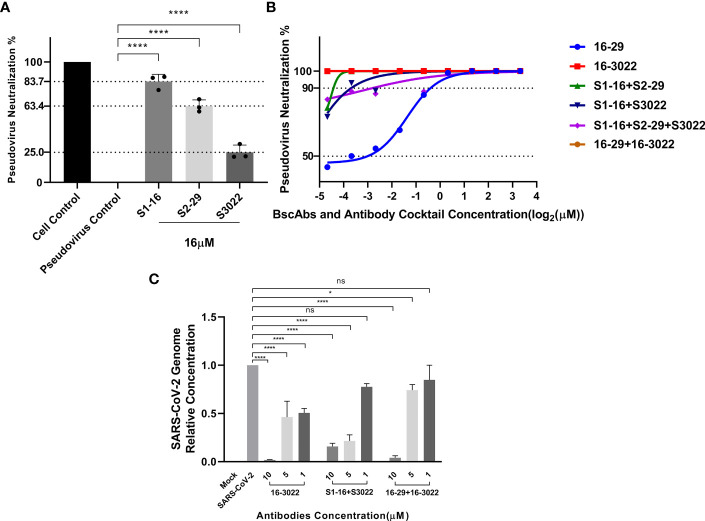
Antiviral activity of antibodies or a cocktail of antibodies against SARS-CoV-2 pseudovirus and SARS-CoV-2(BJ01). **(A)** Neutralization potency of scFvs at the concentration of 16 μM was calculated on the basis of the pseudotyped SARS-CoV-2 neutralization assay (luciferase); **(B)** Neutralization potency of two-fold serial diluted BscAbs from 10 μM and a cocktail of antibodies was calculated on the basis of the pseudotyped SARS-CoV-2 neutralization assay (luciferase). Red and brown lines denote 16-3022 and 16-29 + 16-3022 with IC100 < 39 nM. Wathet blue, green, blue and purple lines denote 16-29, S1-16+S2-29, S1-16+S3022 and S1-16+S2-29+S3022 with IC_90 = _0.76, 0.05, 0.08 and 0.14 μM, respectively; **(C)** Percent inhibition of SARS-CoV-2(BJ01) replication was shown by 16-3022, S1-16+S3022 and 16-29 + 16-3022 in Vero E6 cells. Replication was measured *via* quantification of the viral RNA level using RT-qPCR. Each concentration in the image above represents the mean value of three separate experiments. p ≤ 0.05 (*); p ≤ 0.01 (**); p ≤ 0.001 (***); p ≤ 0.0001 (****); ns, not statistically.

To assess the neutralization ability of scFvs, BscAbs and the antibody cocktail therapy *in vitro*, we initially employed a SARS-CoV-2 pseudovirus that carries wild-type SARS-CoV-2 Spike protein to perform a pseudovirus neutralization test. Consistent with our theorized expectations, all the three scFvs exhibited low neutralization ability against pseudovirus at IC_90_ lower than 16 µM (**Figure 4A**), which may be attributed to their single antigen binding site. However, BscAbs and the cocktail of scFvs (equal molar quantity of S1-16 mixed with S2-29 or S3022) demonstrated much better neutralizing ability than scFv alone and the IC_100_ of BscAb 16-3022 and the cocktail of 16-3022 in combination with 16-29 was lower than 39 nM (**Figure 4B**), which reached a similar concentration level as its parental IgG (Brouwer et al., 2020). These results revealed that different scFvs could cover different regions of SARS-CoV-2 RBD and synergistically enhance the neutralization effectiveness.

### 3.5 *In vitro* inhibition of the genome replication of SARS-CoV-2 original strain and Omicron variant

**Figure 5:**
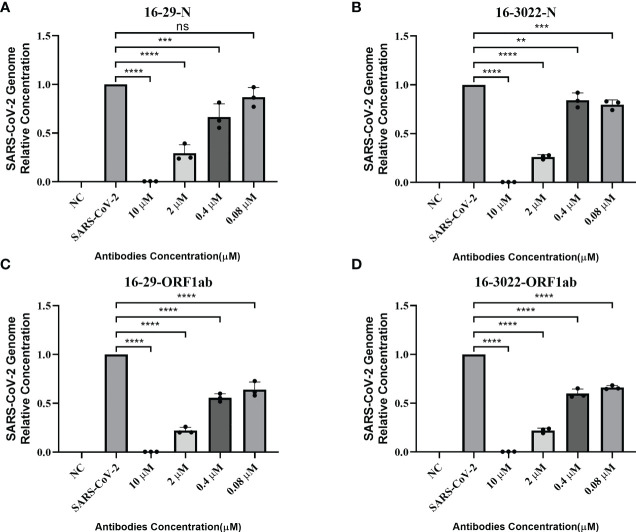
Antiviral activity of BscAb 16-29 and 16-3022 against SARS-CoV-2 Omicron BA.5. **(A-D)** Percent inhibition of SARS-CoV-2 Omicron variant (BA.5) replication was shown by 16-29 and 16-3022 in Vero E6 cells. **(A, B)** Percentage of N gene copy number decrease; **(C, D)** Percentage of ORF1ab gene copy number decrease. Replication was measured *via* quantification of the viral RNA level using RT-qPCR. Each concentration in the image above represents the mean value of three separate experiments. p ≤ 0.05 (*); p ≤ 0.01 (**); p ≤ 0.001 (***); p ≤ 0.0001(****); ns, not statistically.

According to the results of pseudovirus neutralization assay, we selected BscAb S16-3022, a cocktail of S1-16 in combination with S3022, and a cocktail of 16-29 in combination with 16-3022, which exhibited better performance in the pseudovirus neutralization assay, to perform an authentic SARS-CoV-2 nucleic acid replication inhibition assay. RT-qPCR was performed to assess the inhibitory activity of the three candidates on SARS-CoV-2 gene expression. As shown in **Figure 4C**, the three candidates at the concentration of 10 µM effectively inhibited viral replication by up to approximately 98%, 83% and 95%, respectively, compared to the normal cell control group. To investigate whether BscAbs could effectively neutralize SARS-CoV-2 variants, Omicron BA.5 was selected as the test strain for RT-qPCR experiment. As shown in **Figure 5**, both of BscAb 16-29 and 16-3022 at a concentration of 10 µM effectively inhibited viral N or ORF1ab gene replication by more than 99%, compared to the normal cell control group. The reduction in viral gene copy number indicated the therapeutic effect of the BscAbs on SARS-CoV-2 original strain and Omicron variant infection.

### 3.6 Structural basis for enhancing neutralization activity of BscAbs

**Figure 6:**
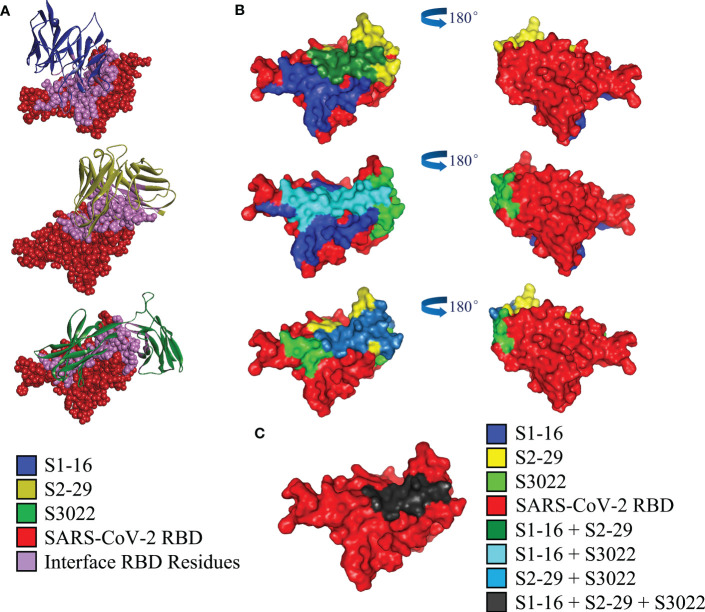
Computerized simulation to reveal the regions of SARS-CoV-2-RBD bound by scFvs. **(A)** Crystal structure of SARS-CoV-2 receptor binding domain in complex with scFvs using molecular docking. S1-16, S2-29 and S3022 are labeled blue, orange and green, respectively; RBD is labeled red, and the bonding interface of RBD to scFvs is labeled purple; **(B)** Molecular docking predicted regions of SARS-CoV-2-RBD that binding to S1-16, S2-29 and S3022 are shown as blue, yellow and light green, respectively; green, dark slate blue and light blue represents the overlap of S1-16 and S2-29, S1-16 and S3022, S2-29 and S3022, respectively; **(C)** The overlap of all three scFvs is marked in dark grey.

**Figure 7:**
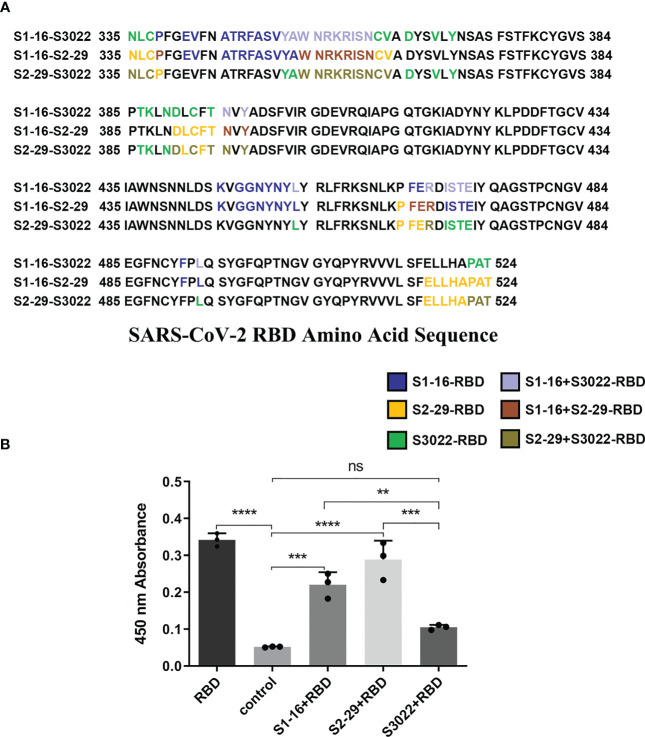
Site coincidence analysis and competitive ELISA validation between S3022 and other scFvs. **(A)** Site coincidence analysis between S3022 and other scFvs on RBD amino acid sequence. The site coincidence between S3022 and S1-16 in RBD is 33.9%; the site coincidence between S3022 and S2-29 in RBD is 47.8% (ratio of the number of coincident amino acid residues to the total number of interacting amino acid residues); **(B)** The competitive ELISA revealed the reaction of S3022 as a coating substrate to RBD bound by three excess scFvs. Each concentration of scFv in the image above represents the mean value of three separate experiments. p ≤ 0.05 (*); p ≤ 0.01 (**); p ≤ 0.001 (***); p ≤ 0.0001 (****); ns, not statistically.

To investigate the structural basis underlying the enhancement of neutralizing activity of BscAbs, DS Protein Docking method was used. Following the ZDOCK program, we obtained optimized 3D structures, as depicted in **Figure 6A**. By analyzing the regions of the RBD that bind to different scFvs (**Figure 6B**), we identified three distinct binding regions that did not overlap precisely. The region of RBD that binds to all three scFvs was marked dark gray (**Figure 6C**), which may be the key conserved region of RBD binding antibody molecules. We then calculated the overlap ratio of amino acid residues binding to RBD between the three scFvs. The site coincidence between S3022 and S1-16 binding to RBD was 33.9% and the value between S3022 and S2-29 was 47.8% (**Figure 7A**).

### 3.7 Epitopes verified by competitive ELISA

To validate the molecular docking results, we conducted a competitive ELISA assay. Using a scFv-RBD-scFv sandwich ELISA, mouse Fc-fused SARS-CoV-2 RBD in S1-16-RBD-S3022 or S2-29-RBD-S3022 sandwich complex was detected by HRP-conjuncted goat anti-mouse secondary antibody. As shown in **Figure 7B**, the S1-16-RBD or S2-29-RBD complex could still bind to S3022 substrate, consistent with the overlap analysis of amino acid sites bound by different scFvs on SARS-CoV-2 RBD. The difference in binding epitopes with other scFvs on RBD render S3022 playing a synergistic role with S1-16 or S2-29 to jointly bind SARS-CoV-2 RBD, thus improving the neutralizing activity of BscAbs.

## 4 Discussion

During the past two decades, novel human coronaviruses have emerged, causing significant loss of life and economic damage(Ludwig and Zarbock, 2020). Over years of research accumulation, monoclonal antibodies are considered as one of the most promising immunotherapeutic agents for the treatment of cancers and epidemic (Zhu et al., 2013; Ying et al., 2014a; Ying et al., 2014b; Ying et al., 2015). However, The efficacy of existing vaccines and therapeutic antibodies are threatened due to the emergence of SARS-CoV-2 variants of concern such as Delta and Omicron variants, which underscores the need for additional antibody-based therapies that potently neutralize variants by targeting multiple sites of SARS-CoV-2 spike protein simultaneously. Combined with the advantage of cocktail and single-molecule strategies, bispecific antibodies (BsAbs) are emerging as an important and promising component of the next generation of therapeutic or neutralizing antibodies to enhance neutralizing activity or prevent virus variant escape. From 1997 to 2020, there were 272 clinical trials of BsAbs studies worldwide, with most concentrated in phase I (n=161), and a limited number of phase I/II, II and III trials (Zhang et al., 2021). Researches on the mechanism of BsAb are mainly focused on T-cell recruitment and double immune checkpoint blocking (Hipp et al., 2020; Hosseini et al., 2021; Kamakura et al., 2021), while research on anti-virus applications is rare.

In this study, we produced and characterized three SARS-CoV-2 RBD-targeted scFvs S1-16, S2-29 and S3022, and two SARS-CoV-2 RBD-targeted BscAbs, 16-29 and 16-3022. We aimed to explore whether CR3022, which exhibited synergistic effects with other SARS-CoV RBD-targeted Nabs during the SARS epidemic (Yuan et al., 2020), could also be compatible with other SARS-CoV-2 RBD-targeted antibodies to enhance neutralizing activity. Our results revealed that neither S3022 nor S1-16 exhibited effective neutralizing activity in the pseudotyped virus neutralization assay whereas 16-3022 showed a nanomolar level of EC_90_ lower than 39 nM at the same concentration. Additionally, 16-3022 exhibited a better EC_90_ against pseudotyped virus than 16-29, which is made up of two SARS-CoV-2 RBD-targeted scFvs, despite their RBD-targeted affinity being close (**Figure 1C**). These findings suggest that S3022 has the potential to be used as a co-operative antibody to construct high neutralizing activity BscAbs or in combination with other antibodies for the use of cocktail therapy against SARS-CoV-2. To further verify the effective inhibition of BscAb or cocktail therapy against authentic virus, we determined the inhibition of the virus genome copy of the three antibodies or combinations of antibodies that exhibited better performance in the pseudotyped virus neutralization assay. The results of RT-qPCR showed that the virus genome replication was inhibited by up to approximately 98%, 83% and 95% under the treatment of 16-3022, the cocktail of S1-16 in combination with S3022 and the cocktail of 16-3022 in combination with 16-29 at a concentration of 10 μM compared with the control group, respectively. Furthermore, the effective neutralizing activity of the BscAbs against the Omicron variant suggests that this form of antibody has the potential to combat the escape of the virus. Additionally, we also noticed that although all three scFvs exhibited poor neutralization activity, the neutralizing activity was significantly increased when they were combined in pairs. Based on this, we speculated that although they are all SARS-CoV-2 RBD-targeted scFvs, the antigenic epitopes of the RBD they are binding to are not completely identical. To gain insight of this phenomenon, we simulated the binding regions of scFvs to SARS-CoV-2 RBD using homologous modeling and molecular docking techniques, and the results showed that they did not completely overlap. The results of molecular docking simulation were verified by competitive ELISA, in which S3022 could still bind to S1-16-RBD or S2-29-RBD complex.

It is important to acknowledge several additional limitations of this study. First, *in vitro* neutralizing activity can poorly predict *in vivo* neutralizing activity of the antibodies, further *in vivo* challenge protection tests are needed to characterize the effective neutralizing activity of them. Second, it could be more accurate and reliable to use the complete S protein containing the RBD tripolymer which is closer to the natural structure of the SARS-CoV-2 as the molecular docking model. Third, More experiments need to be designed to characterize physicochemical properties and safety of antibody molecules, such as thermal stability and whether they can cause inflammatory responses *in vivo*.

In summary, our results revealed the robust neutralizing activity of BscAb 16-29 and 16-3022 against SARS-CoV-2 and the highly transmissible Omicron variant. Furthermore, we have demonstrated the synergistic potential of the SARS-CoV RBD-targeting scFv S3022 with other SARS-CoV-2 RBD-targeting antibodies, which can concurrently recognize distinct epitopes on the RBD, thereby amplifying the neutralization potency. This innovative approach offers a promising avenue for the development of subsequent antibody therapies against SARS-CoV-2. Combining the advantages of cocktails and single-molecule strategies, BscAbs hold great promise in serving as efficacious prophylactic and therapeutic agents, and may find wide application in treating SARS-CoV-2 or other viral diseases infection and escape.

## Data availability statement

The original contributions presented in the study are included in the article/**Supplementary Material**. Further inquiries can be directed to the corresponding authors.

## Author contributions

Conceptualization: NX and WL; data curation: YW; formal analysis: MD; funding acquisition: WL; investigation: XW and GL; methodology: BL; project administration: NX; resources: JZ; software: CS; visualization: CS; writing – original draft: KY and HY; writing – review and editing: NX. All authors contributed to the article and approved the submitted version.
