# Corrigendum: A neutralizing bispecific single-chain antibody against SARS-CoV-2 Omicron variant produced based on CR3022

**DOI:** 10.3389/fcimb.2023.1264974

**Published:** 2023-08-24

**Authors:** Kaikai Yu, Bin Liu, Haotian Yu, Chengbiao Sun, Xuefeng Wang, Guorui Li, Mingxin Dong, Yan Wang, Jianxu Zhang, Na Xu, Wensen Liu

**Affiliations:** ^1^ Changchun Veterinary Research Institute, Chinese Academy of Agricultural Science, Changchun, Jilin, China; ^2^ Academic Affairs Office, Jilin Medical University, Jilin, Jilin, China; ^3^ State Key Laboratory of Respiratory Disease, National Clinical Research Center for Respiratory Disease, Guangzhou Institute of Respiratory Health, The First Affiliated Hospital of Guangzhou Medical University, Guangzhou, Guangdong, China; ^4^ College of Life Sciences and Food Engineering, Inner Mongolia Minzu University, Tongliao, China

**Keywords:** SARS-CoV-2, COVID-19, single-chain variable fragment, bispecific antibody, Omicron variant

## Error in Author List

In the published article, several of the authors were labeled with the wrong affiliation. The corrected author list appears below.

Kaikai Yu^1†^, Bin Liu^3†^, Haotian Yu^1†^, Chengbiao Sun^1^, Xuefeng Wang^1^, Guorui Li^4,^ Mingxin Dong^1^, Yan Wang^1^, Jianxu Zhang^1^, Na Xu^2^* and Wensen Liu^1*^

The authors apologize for this error and state that this does not change the scientific conclusions of the article in any way. The original article has been updated.

